# Asymmetric Somatic Hybridization Affects Synonymous Codon Usage Bias in Wheat

**DOI:** 10.3389/fgene.2021.682324

**Published:** 2021-06-11

**Authors:** Wenjing Xu, Yingchun Li, Yajing Li, Chun Liu, Yanxia Wang, Guangmin Xia, Mengcheng Wang

**Affiliations:** ^1^The Key Laboratory of Plant Development and Environmental Adaption, Ministry of Education, School of Life Science, Shandong University, Jinan, China; ^2^Shijiazhuang Academy of Agriculture and Forestry Sciences, Shijiazhuang, China

**Keywords:** introgression line, genomic shock, synonymous codon usage bias, somatic hybridization, wheat

## Abstract

Asymmetric somatic hybridization is an efficient strategy for crop breeding by introducing exogenous chromatin fragments, which leads to whole genomic shock and local chromosomal shock that induces genome-wide genetic variation including indel (insertion and deletion) and nucleotide substitution. Nucleotide substitution causes synonymous codon usage bias (SCUB), an indicator of genomic mutation and natural selection. However, how asymmetric somatic hybridization affects SCUB has not been addressed. Here, we explored this issue by comparing expressed sequence tags of a common wheat cultivar and its asymmetric somatic hybrid line. Asymmetric somatic hybridization affected SCUB and promoted the bias to A- and T-ending synonymous codon (SCs). SCUB frequencies in chromosomes introgressed with exogenous fragments were comparable to those in chromosomes without exogenous fragments, showing that exogenous fragments had no local chromosomal effect. Asymmetric somatic hybridization affected SCUB frequencies in indel-flanking sequences more strongly than in non-flanking sequences, and this stronger effect was present in both chromosomes with and without exogenous fragments. DNA methylation-driven SCUB shift was more pronounced than other SC pairs. SCUB shift was similar among seven groups of allelic chromosomes as well as three sub-genomes. Our work demonstrates that the SCUB shift induced by asymmetric somatic hybridization is attributed to the whole genomic shock, and DNA methylation is a putative force of SCUB shift during asymmetric somatic hybridization. Asymmetric somatic hybridization provides an available method for deepening the nature of SCUB shift and genetic variation induced by genomic shock.

## Introduction

During domestication and improvement processes, the genetic base and diversity of crops gradually become low. The wild relatives of crops retain genetic diversity, and therefore are a valuable genetic resource for crop breeding. The genetic materials of wild relatives can be introduced intro crops *via* remote sexual hybridization and somatic hybridization (Tanksley and McCouch, 1997; Zamir, 2001). Somatic hybridization comprises symmetric and asymmetric somatic hybridization (Xia, 2009). Symmetric somatic hybridization is that protoplasts of two species are fused to form fused cells, and fused cells regenerate into seedlings. Asymmetric somatic hybridization is that protoplasts of one species (donor) are irradiated by UV to fragment the genome prior to fusion and then irradiated protoplasts are fused with unirradiated protoplasts (recipient) (Xia, 2009). During asymmetric somatic hybridization, very small amounts of chromatin fragments of the donor are introgressed into the recipient genome (Wang et al., 2004; Cui et al., 2015); thus, asymmetric somatic hybrid is a special allopolyploidy. The transient co-emergence of donor and recipient genomes in fused cell as well as the introgression of exogenous chromatin segments into the genome lead to a strong genomic shock. Similar to diploydization of polyploydies (McClintock, 1984; Chen, 2007), the genomic shock can induce genome-wide genetic variation [mainly insertion and deletion (indel) and single-nucleotide substitution] in somatic hybrids (Liu and Xia, 2014).

Single-nucleotide substitution is a major force of evolution. Single-nucleotide substitution in protein-coding sequences produces either a synonymous codon (SC) or a non-synonymous codon. Except for methionine and tryptophan, all amino acids are encoded by at least two SCs. SCs of an amino acid appear to have a different frequency in the genome, which is referred to as synonymous codon usage bias (SCUB). Nucleotide substitution between SCs is generally considered to be functionally neutral (King and Jukes, 1969; Nei and Gojobori, 1986). In fact, SCs affect recombination rates, splicing regulation, transcription efficiency, RNA secondary structure, mRNA stability, translational efficiency, and accuracy in the regulation of gene expression, protein folding, and so on (Marais et al., 2001; Warnecke and Hurst, 2007; Zhang et al., 2009; Tuller et al., 2010; Presnyak et al., 2015). Thus, SCUB has proved to reflect the mutation, genetic drift, and natural selection (Akashi and Eyre-Walker, 1998; Akashi, 2001; Guo and Yuan, 2009; Wang Z. et al., 2014) and to be closely associated with plant evolution (Qin et al., 2013; Qi et al., 2015; Xu et al., 2015). The induction of genome-scale genetic variation induced by asymmetric somatic hybridization is under selection pressure in wheat (Wang et al., 2018). Given that the nucleotide substitution between SCs suffers from less selection pressure than the substitution between non-SCs, the interesting question is that whether asymmetric somatic hybridization affects SCUB in the recipient genome.

Unlike allopolyploidy that the genomes of progenitors stably co-emerge in the nucleus, the recipient genome is introgressed several donor chromatin fragments in asymmetric somatic hybrids. In wheat, the introgressed exogenous fragments results in both whole genome shock and local chromosomal shock, which induces genome-scale genetic variation and nucleotide substitution in indel-flanking sequence, respectively, in a non-random manner (Wang et al., 2018). This indicates that the introgression of exogenous fragments has an effect on genetic variation in chromosomes possessing chromatin fragments. However, whether the introgressed fragments influence SCUB through local chromosomal shock has not been addressed.

We previously bred a common wheat cultivar Shanrong 3 (SR3) *via* asymmetric somatic hybridization with the wheat cultivar Jinan 177 (JN177) as the recipient and tall wheatgrass (*Thinopyrum elongatum*, wheat's close relative) as the donor (Xia et al., 2003). SR3 genome possesses six exogenous fragments (Wang et al., 2005; Liu et al., 2015) and occurs in genome-scale genetic variation including nucleotide substitution and insertion and deletion (indel) (Feng et al., 2004; Liu et al., 2007, 2009, 2015; Wang et al., 2015, 2018), which contributes to salt tolerance of SR3. Thus, it is necessary to uncover whether this genetic variation was along with SCUB alteration, which will provide evidence for further understanding the characteristics of genetic variation induced by asymmetric somatic hybridization, and how genetic variation accounts for the salt tolerance of SR3 as well as the change in traits of introgression lines. Here, we used the unigenes of SR3 and JN177 (Wang et al., 2015) and found that asymmetric somatic hybridization caused SCUB shift, and introgressed fragments had no stronger effect on SCUB in local chromosome.

## Materials and Methods

### The Introduction of Wheat Introgression Cultivar Shanrong 3

The common bread wheat cultivars Jinan 177 (JN177) and Shanrong 3 (SR3) were used for analysis (Wang et al., 2018), both of which were bred by our lab (Dr. Guangmin Xia) and have not been deposited in a publicly available herbarium. The detail of JN177 and SR3 was introduced in our previous study (Xia et al., 2003). Briefly, SR3 is a wheat cultivar with high salt and drought tolerance bred *via* asymmetric hybridization with the common wheat cultivar JN177 (modest salt and drought tolerance) as the recipient and wheat's close relative tall wheatgrass (*T. elongatum*, topmost salt tolerance) as the donor, and SR3 genome is introgressed with several chromatin fragments of the donor (Xia et al., 2003). The genome of SR3 took place with genome-wide genetic and epigenetic variation (Liu et al., 2015; Wang et al., 2015, 2018).

### EST Sequencing, Chromosomal Localization, and Genetic Variation Analysis

The methods of expressed sequence tag (EST) sequencing, chromosomal localization, and genetic variation analysis were present in the previous study. Briefly, the ESTs of JN177 and SR3 were used for constructing cDNA library, which were used for large-scale EST sequencing with the Sanger sequencing method. After sequence cleaning, highly qualified EST sequences (>100 nt) were assembled to produce unigenes, and PCR was conducted to confirm the quality of unigene assembly. The chromosomal localization was determined by BLASTing the unigenes against wheat survey database (http://wheat-urgi.versailles.inra.fr/Seq-Repository). The local BLASTN was carried out to analyze the genetic variation between the unigenes of SR3 and JN177. The local BLASTX against the non-redundant protein database was performed to extract CDS of unigenes.

### SCUB Frequency Calculation

The frequency of a SC was calculated by the ratio of the amount of this codon to the amount of 59 SCs encoding 18 amino acids except for three stop codons TAA, TAG and TGA, ATG (methionine) and TGG (tryptophan) in CDS of all unigenes according to our previous study [8]. The SCUB frequency of a given amino acid was defined as the amount of SCs with C and G at the third position to the amount of SCs with A and T at the third position in CDS of all unigenes. For instance, alanine is coded by GCA, GCT, GCC, and GCG, so the SCUB frequency of alanine was defined as the ratio of GCC and GCG amount to GCA and GCT amount. Total SCUB frequency was defined as the ratio of the amount of all SCs with A, T, C, or G at the third position (NNA, NNT, NNC, or NNG) to the amount of all SCs in CDS of all unigenes.

DNA methylation is a major source of DNA variation in the nuclear genome, given that methylated cytosine (5 mC) is readily converted into thymine [21]. Methylation is mainly present in C of CpG, and the conversion of 5 mC produces TpG in sense strand and CpA in antisense strand. Given the lower selection pressure on the third position of codons, the conversion of NCG to NCA (the second-third position) as well as NC|G to NT|G (the third-next codon's first position) would be dominant, which leads to the bias to A- and T-ending codons [6]. Thus, the ratios of NXA/NXG (X = A, T, C, or G) can reflect the effect of the second nucleotide on the conversion from G to A at the third positon, and the ratios of NT|X/NG|X (X = A, T, C, or G at the first position of the next codon) can reflect the first nucleotide of the next codon on the conversion from C to T at the third position. If DNA methylation contributes to SCUB, the ratio of NCA/NCG would be higher than those of NXA/NXG (X = A, G, T), and the ratio of NT|G/NC|G would be higher than those of NT|X/NG|X (X = A, T, C). Based on this, the difference between the ratios of NCA/NCG and NAA/NAG, NGA/NGG, and NTA/NTG as well as the difference between the ratios of NT|G/NC|G and NT|A/NC|A, NT|C/NC| NT|G/NC|G, and NT|T/NG|T were calculated to assess the potential association between DNA methylation and SCUB.

The association between DNA methylation and SCUB was also analyzed using the ratio of NXA/NXG of amino acids (Ala, Arg, Gln, Glu, Gly, Leu, Lys, Pro, Ser, Thr, and Val) that are encoded by SCs including A and G at the third position. The ratio of NXA/NXG ratio of SR3 to NXA/NXG ratio of JN177 was used to access the putative contribution of DNA methylation to the change in SCUB induced by asymmetric somatic hybridization.

### Indel-Flanking and Indel-Remote Sequence Extraction

Fifteen codons (45 nt) of 5′- and 3′-flanking sequences of indels (insertions and deletions) were extracted for calculating SCUB frequency. The indels with instance >45 nt from start and stop codons were used for analysis to avoid the terminal effect. Sequences with length <90 nt between two indels were not considered as flanking sequences to avoid the effect of adjacent indels. The sequences with distance >45 nt from indels were extracted as non-flanking sequences.

### Statistical Analysis

The chi square (χ^2^) test of the cross-table analysis was performed to establish the significance of differences in the SCUB frequency based on SCs in CDS sequences of all unigenes, mapped unigenes, introgressed unigenes, and non-introgressed unigenes between SR3 and JN177. The difference in SCUB frequencies of each of the 18 amino acids between SR3 and JN177 was performed using the amount of A- and T-ending SCs and C- and G-ending SCs of a given amino acid. The difference in total SCUB frequencies between SR3 and JN177 was performed using the amount of all SCs with A, T, C, or G at the third position (NNAs, NNTs, NNCs, or NNGs). The significance of differences in SCUB frequency related to the third nucleotide position concerning DNA methylation between SR3 and JN177 was also analyzed with the χ^2^ test of the cross-table analysis. For example, the difference in NCA/NCG ratio (the second–third nucleotide combination) between SR3 and JN177 was analyzed by the amounts of NCA and NCG of SR3 and JN177; the difference in NT|G/NC|G ratio (the third nucleotide and the first nucleotide of next codon combination) between SR3 and JN177 was analyzed by the amounts of NT|G and NC|G of SR3 and JN177. Besides, the difference of SCUB frequencies in other comparisons such as those between introgressed and non-introgressed sequences as well as those between indel 5′-flanking and 3′-flanking sequence were also conducted using the χ^2^ test of the cross-table analysis. The difference in NXA/NXG SCs of an amino acid encoding by A- and G-ending SCs (Ala, Arg, Gln, Glu, Gly, Leu, Lys, Pro, Ser, Thr, and Val) between SR3 and JN177 was calculated with the χ^2^ test of the cross-table analysis, and the amounts of NXA and NXG of SR3 and JN177 were used for calculation. The difference in the ratios of NCG/NCA of Ala, Pro, Ser, and Thr between SR3 and JN177 from the ratios of N(G/T)G/N(G/T)A of Arg, Gln, Glu, Gly, Leu, Lys, and Val between SR3 and JN177 was calculated with the *t*-test. The fluctuation was assessed by the coefficient of variation (CV), which is calculated as the ratio of standard deviation to mean. The consistency of SCUB frequency was detected *via* reliability analysis (model was set as alpha), and Cronbach's alpha value was used to indicate the consistency.

## Results

### SCUB Appeared to Be Different Between Shanrong 3 and Jinan 177

We previously large-scale sequenced the cDNA libraries and obtained 9,634 and 7,107 unigenes from Shanrong 3 (SR3) and Jinan 177 (JN177), respectively (Wang et al., 2015). SR3 genome took place at a high frequency of genetic variation such as nucleotide substitution (Liu et al., 2015; Wang et al., 2015, 2018); therefore, we analyzed here to know the SCUB alteration. Approximately 77.1% of SR3 and 79.2% of JN177 in length were coding sequences (CDS) (Supplementary Figure 1A), which included ~1.4 × 10^6^ and 1.3 × 10^6^ codons (Supplementary Figure 1B). Among the 59 SCs that encode 18 amino acids, the frequencies of both codons with A/T at the third position (NNAs/NNTs) and NNCs/NNGs were various, and NNCs and NNGs were more dominant than NNAs and NNTs; the frequency of each of the codons was comparable among JN177 and SR3 (Supplementary Figure 2).

**Figure 1:**
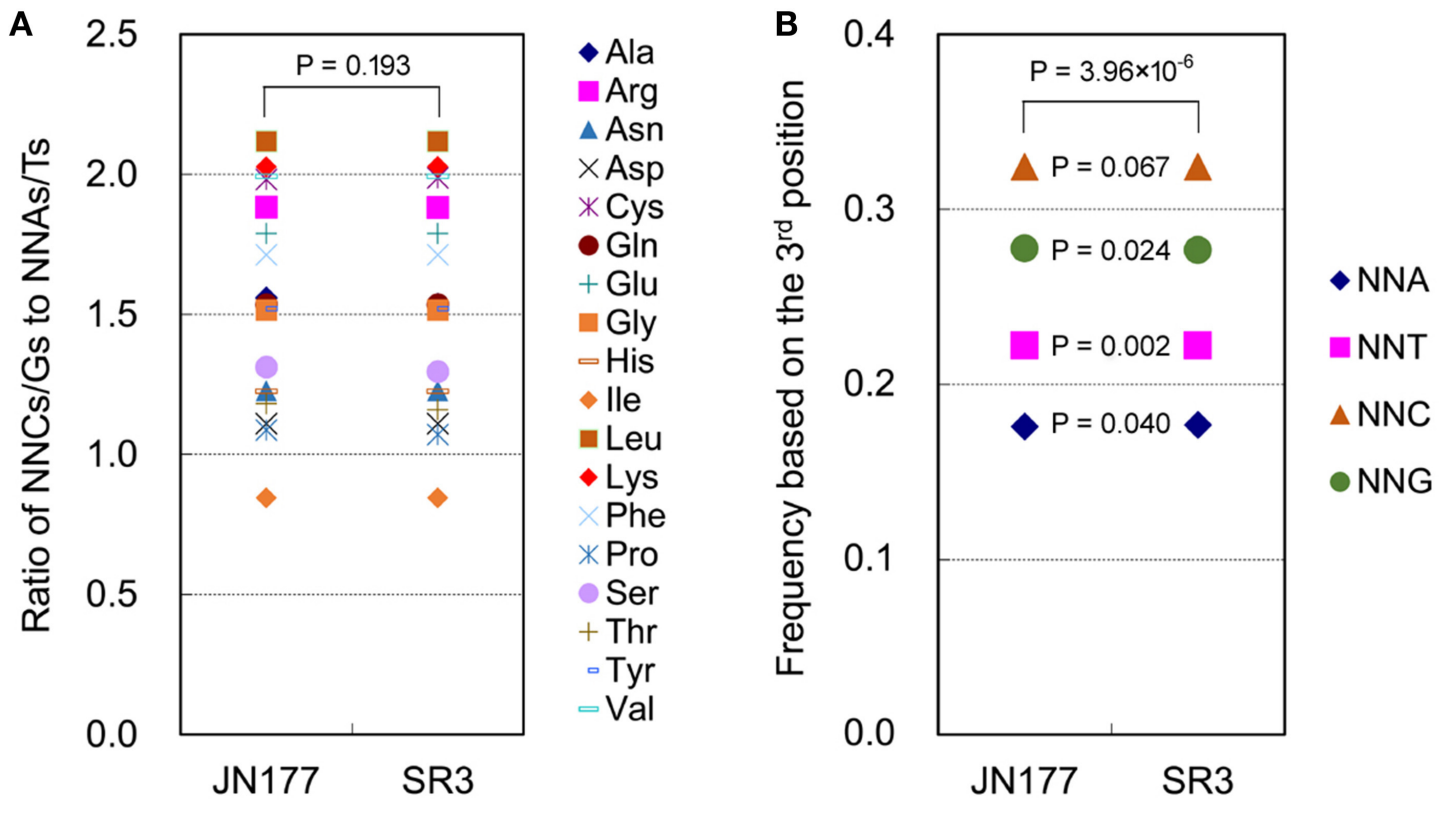
SCUB was shifted in the genome of SR3. **(A)** The ratios of the frequencies of NNC/NNG SCs to NNA/NNT SCs. NNCs/Gs: SCs with C or G as their final bases; NNAs/Ts: SCs with A or T as their final base. **(B)** The total frequency of NNA, NNT, NNC, and NNG codons. The total frequency was calculated as the ratio between the number of all SCs ending with A, T, C, or G and the amount of all SCs. The statistical comparison was conducted with chi square (χ^2^) test of cross-table analysis.

SCUB frequency of a given amino acid encoded by SCs was calculated as the ratio of NNCs/NNGs amount to NNAs/Ts amount (Figure 1A). The SCUB frequencies of 18 amino acids were 0.844 to 2.118 in JN177 and SR3, and they were different among each other [coefficient of variation (CV) = 0.069–0.501] (Supplementary Table 1). The SCUB frequencies of amino acids was almost comparable between two cultivars (Figure 1A), except that the frequencies of Ala, Pro, Ser, and Thr were different between JN177 and SR3 (*P* < 0.05, χ^2^ test) (Supplementary Table 1).

Total SCUB frequencies of four types of codons with A, T, C, and G at the third positon (NNA, NNT, NNC, and NNG), calculated as the ratios of the amounts of four types of codons to the amount of all codons, were used to compare SCUB between JN177 and SR3. NNC and NNG were more pronounced than NNA and NNT (Figure 1B). Generally, the frequencies of NNA, NNT, NNC, and NNG showed a significant difference between JN177 and SR3 (*P* = 3.96 × 10^−6^, χ^2^ test). In comparison with JN177, SR3 had higher frequencies of NNA and NNT (*P* = 0.040 and 0.002), but lower frequencies of NNC and NNG (*P* = 0.024 and 0.067). The results indicate that asymmetric somatic hybridization affected SCUB.

### Introgression of Exogenous Fragments had No Genome-Wide Effect on SCUB

**Figure 2:**
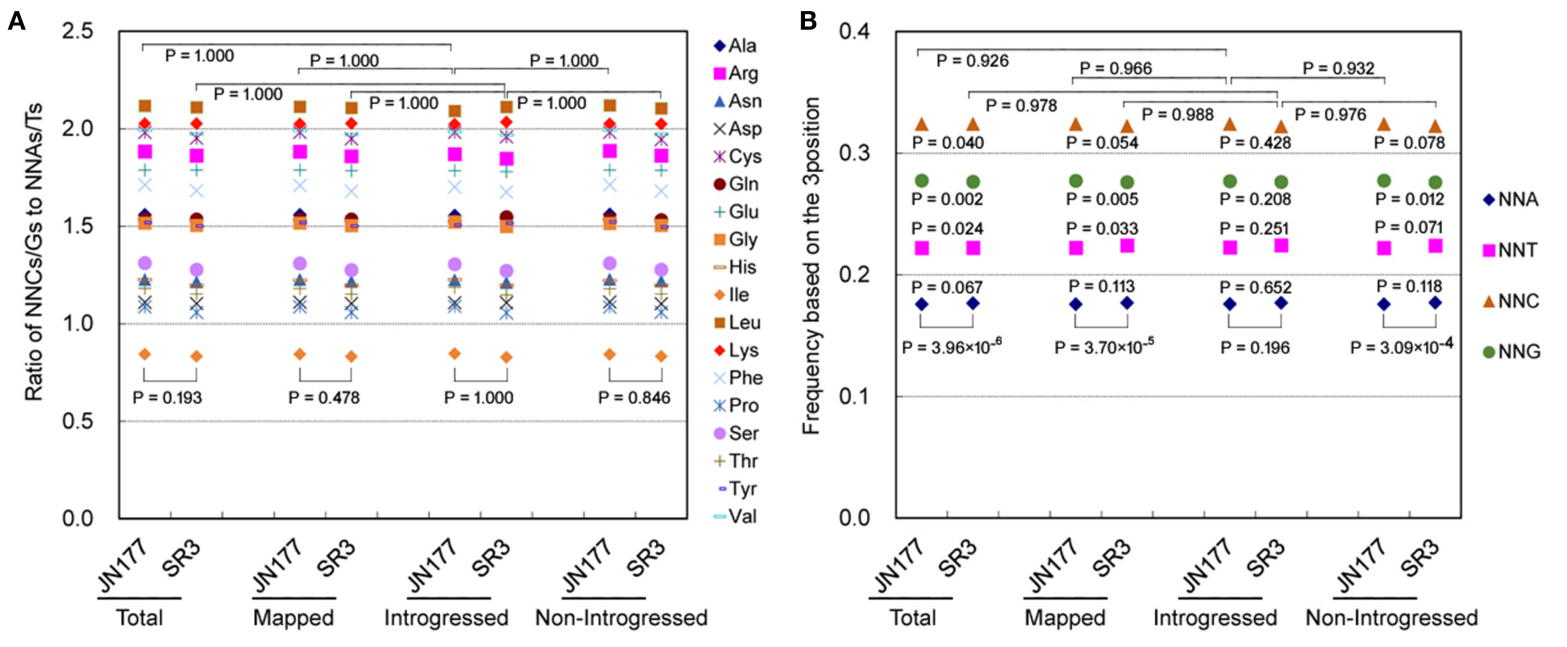
SCUB was shifted to a similar extent in chromosomes introgressed with and without exogenous fragments. **(A)** The ratios of the frequencies of NNC/NNG SCs to NNA/NNT SCs. NNCs/Gs: SCs with C or G as their final bases; NNAs/Ts: SCs with A or T as their final base. **(B)** The total frequency of NNA, NNT, NNC, and NNG codons. The total frequency was calculated as the ratio between the number of all SCs ending with A, T, C, or G and the amount of all SCs. Total: all unigenes; Mapped: unigenes mapped to chromosomes; Introgressed: unigenes from chromosomes introgressed with exogenous fragments; Non-introgresed: unigenes from chromosomes without exogenous fragments. The statistical comparison was conducted with chi square (χ^2^) test of fourfold cross-table analysis.

In the genome of SR3, six exogenous fragments were found to be introgressed in chromosomes 1BL, 1DL, 2AL, 2DL, 5BS, and 6DS (Wang et al., 2004). We previously found that genetic variation in these chromosomes had no significant difference to that in the other chromosomes without introgressed fragments (Wang et al., 2018). To know the effect of exogenous fragments on SCUB in introgressed chromosomes, the SCUB frequencies of unigenes mapped to 21 chromosomes were compared. In general, the SCUB frequencies of the amino acids encoded by SCs were similar between JN177 and SR3 when mapped (unigenes mapped to chromosomes), introgressed (unigenes mapped to chromosomes introgressed with exogenous fragments), or non-introgressed (unigenes mapped to chromosomes without exogenous fragments) unigenes were used for calculation, respectively (*P* > 0.05, χ^2^ test), except for Ala and Pro of mapped unigenes (*P* = 0.023 and 0.043, χ^2^ test) (Figure 2A; Supplementary Table 2). However, the total SCUB frequencies of either mapped or non-introgressed unigenes were significantly different between JN177 and SR3 (*P* = 3.70 × 10^−5^ and 3.09 × 10^−4^, χ^2^ test), but the difference was weakened in introgressed unigenes (*P* = 0.196, χ^2^ test) (Figure 2B). Moreover, each of the NNA, NNT, NNC, and NNG frequencies were obviously different between SR3 and JN177 when total, mapped, and non-introgressed unigenes were calculated, and SR3 had higher NNA and NNT ratios but lower NNC and NNG ratios. Although the ratio values were similar to non-introgressed unigenes, the difference in each of the NNA, NNT, NNC, and NNG ratios of introgressed unigenes between JN177 and SR3 were statistically not obvious (*P* = 0.281–0.652, χ^2^ test) (the reason was that the codon amounts of introgressed unigenes were drastically fewer than those of non-introgressed unigenes). The findings showed that SCUB in chromosomes introgressed with and without exogenous fragments was both shifted.

The SCUB frequencies of the amino acids encoded by SCs were generally comparable among total unigenes, mapped unigenes, non-introgressed unigenes, and introgressed unigenes in either JN177 (CV = 6.94 × 10^−5^ to 0.0064, Cronbach's alpha = 1.00) and SR3 (CV = 1.79 × 10^−4^ to 0.0059, Cronbach's alpha = 1.00) (Figure 2A; Supplementary Table 3). The SCUB frequencies of these amino acids in introgressed unigenes had no significant difference from those in non-introgressed unigenes as well as mapped unigenes and total unigenes in either JN177 or SR3 (Figure 2A; Supplementary Table 2). Moreover, the total SCUB frequencies of introgressed unigenes, illustrated by the ratios of NNA, NNT, NNC, and NNG, were also similar to those of non-introgressed unigenes, mapped unigenes, and total unigenes in JN177 (*P* = 0.926–0.966) and SR3 (*P* = 0.976–0.988) (Figure 2B). These results indicated that SCUB in chromosomes introgressed with exogenous fragments was not affected more strongly than SCUB in chromosomes without exogenous fragments.

### SCUB Was Promoted in Indel-Flanking Sequences

We previously found that nucleotide substitution preferred sequences adjacent to indels in SR3 genome (Wang et al., 2018). To clarify whether SCUB had a similar effect, the CDS sequences aligned between JN177 and SR3 were extracted, and sequences flanking to indels between JN177 and SR3 as well as sequences not flanking to indels were used for calculating SCUB frequency. The SCUB frequencies of amino acids encoded by SCs were also similar between introgressed sequences and non-introgressed sequences as well as aligned and mapped sequences (*P* = 1.000, χ^2^ test) (Supplementary Figure 3A; Supplementary Table 4); the total SCUB frequencies were also comparable in these comparison combinations (*P* = 0.123–0.484, χ^2^ test) (Supplementary Figure 3B).

**Figure 3:**
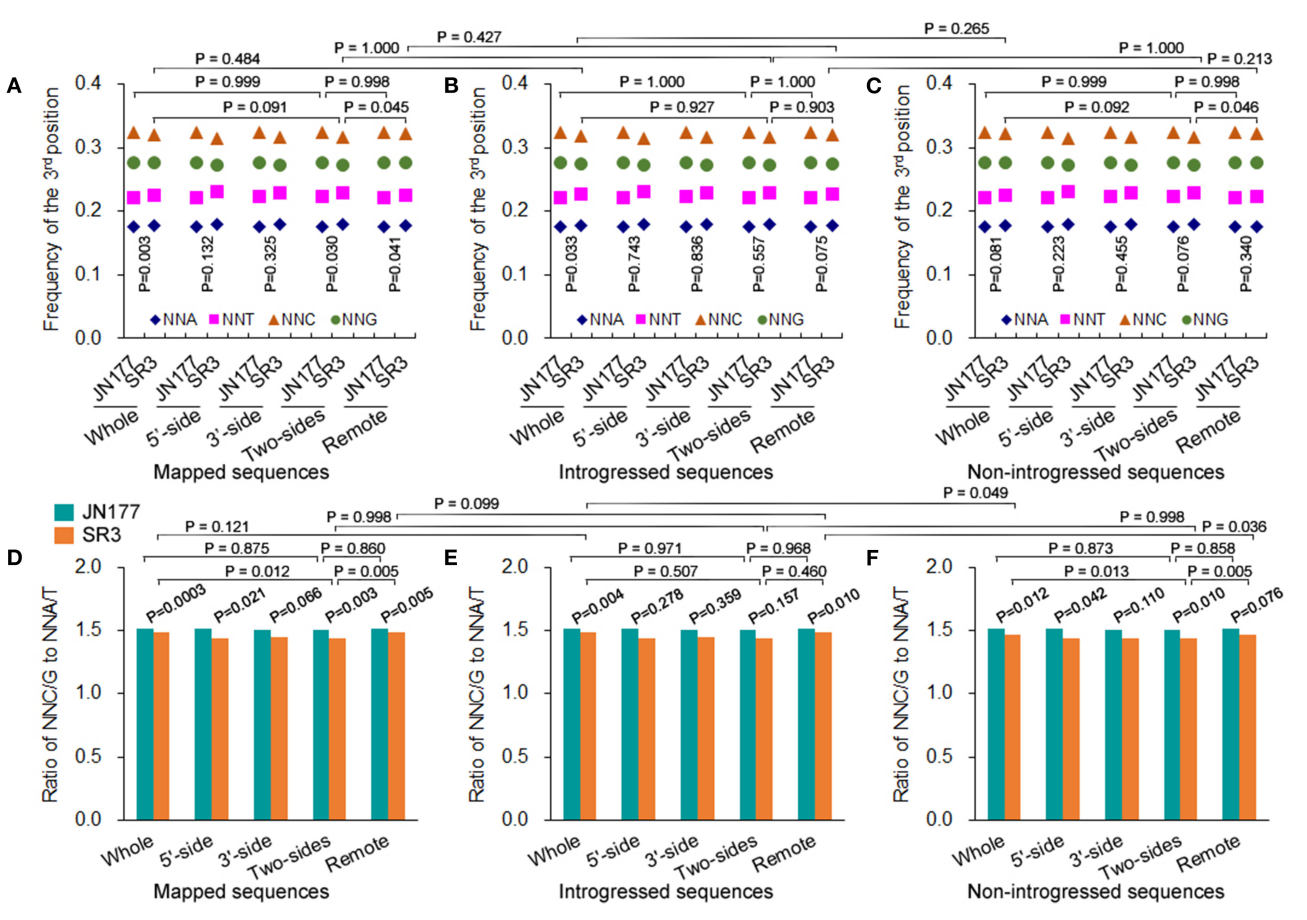
SCUB shift was promoted close to indels. **(A–C)** The total frequencies of NNA, NNT, NNC, and NNG codons in indel-flanking and non-flanking sequences. **(D–F)** The ratio of NNC and NNG frequency to NNA and NNT frequency. Whole: the whole part of sequences; 5′-side: 5′-side sequences flanking to indels; 3′-side: 3′-side sequences flanking to indels; Two-sides: 5′- and 3′-side sequences flanking to indels; Remote: the sequences non-flanking to indels. The statistical comparison was conducted with chi square (χ^2^) test of fourfold cross-table analysis.

The total SCUB frequencies of flanking and non-flanking (remote) sequences of indels were calculated. For mapped sequences, the total SCUB frequencies showed a significant difference between JN177 and SR3 when the whole aligned sequences were compared (*P* = 0.003, χ^2^ test) (Figure 3A). Consistently, the total SCUB frequencies of indel-flanking sequences (two-sides) and non-flanking sequences of SR3 were also obviously different from those of JN177 (*P* = 0.030 and 0.041, χ^2^ test). In comparison with JN177, SR3 had slightly higher frequencies of NNA and NNT but lower frequencies of NNC and NNG in whole and non-flanking sequences, while an increase of NNA and NNT frequencies and a decrease of NNC and NNG frequencies were stronger in SR3 (Figure 3A; Supplementary Table 5). To further evaluate the effect of indels on SCUB, we calculate the ratio of NNC/G to NNA/T (Figure 3D). When compared with JN177, SR3 had significantly lower ratios in whole, indel flanking (5′-, 3′-, and two sides), and non-flanking sequences (*P* = 0.0003–0.066, χ^2^ test). On the other hand, for mapped sequences, the total SCUB frequencies were comparable among whole, indel flanking, and non-flanking sequences in JN177 (Figure 3A). In SR3, the total SCUB frequencies of whole sequences and indel non-flanking sequences were comparable (*P* = 0.927, χ^2^ test), and they were both higher than that of indel-flanking sequences (*P* = 0.091 and 0.045, χ^2^ test); two sides of indel-flanking sequences had similar total SCUB frequency with either 5′- or 3′-side. Consistently, the ratio of NNC/G to NNA/T of indel-flanking sequences was lower than those of whole and non-flanking sequences in SR3 (*P* = 0.012 and 0005, χ^2^ test), as was not found in JN177 (*P* = 0.775 and 0.860, χ^2^ test) (Figure 3D). These data were also found in introgressed sequences and non-introgressed sequences (Figures 3B,C,E,F). Note that, in introgressed sequences, although difference values of the ratios were similar to those of mapped and non-introgressed sequences, some *P*-values of the comparisons were more than 0.05 (Figures 3B,E), which resulted from the SC amount of indel-flanking sequences being drastically fewer than whole and non-flanking sequences. These results indicate that SCUB shift was promoted in indel-flanking sequences.

The total SCUB frequencies of whole sequences were comparable between introgressed sequences and non-introgressed sequences/mapped sequences in SR3 (*P* = 0.265 and 0.484, χ^2^ test) (Figures 3A–C). Similarly, the total SCUB frequencies of both indel-flanking sequences and non-flanking sequences were almost the same in these comparisons. The ratios of NNC/G to NNA/T of indel-flanking, non-flanking, and whole sequences in introgressed sequences were also not different from those in mapped sequences of SR3 (*P* = 0.121, χ^2^ test) (Figures 3D,E). In comparison between introgressed and non-introgressed sequences, the ratios of NNC/G to NNA/T of indel-flanking sequences were similar (*P* = 0.998, χ^2^ test) but those of whole and non-flanking sequences appeared to be slightly different (*P* = 0.049 and 0.036, χ^2^ test) (Figures 3E,F). These data show that the preference of SCUB in the sequences adjacent to indels was not improved in the chromosomes with exogenous fragments.

### The Association Between DNA Methylation and SCUB

Asymmetric somatic hybridization induced genome-scale epigenetic variation (DNA methylation) in SR3 genome (Liu et al., 2015). DNA methylation mediated C-to-T conversion and partially accounts for genetic variation induced by asymmetric somatic hybridization (Wang et al., 2018); thus, we analyzed whether it is also associated with SCUB in SR3 genome. The conversion produces C to T and G to A in sense and antisense strands, respectively, so the conversions lead to the NCG-to-NCA shift (the second–third position) and the NC|G-to-NT|G shift (the third-next codon's first position).

**Figure 4:**
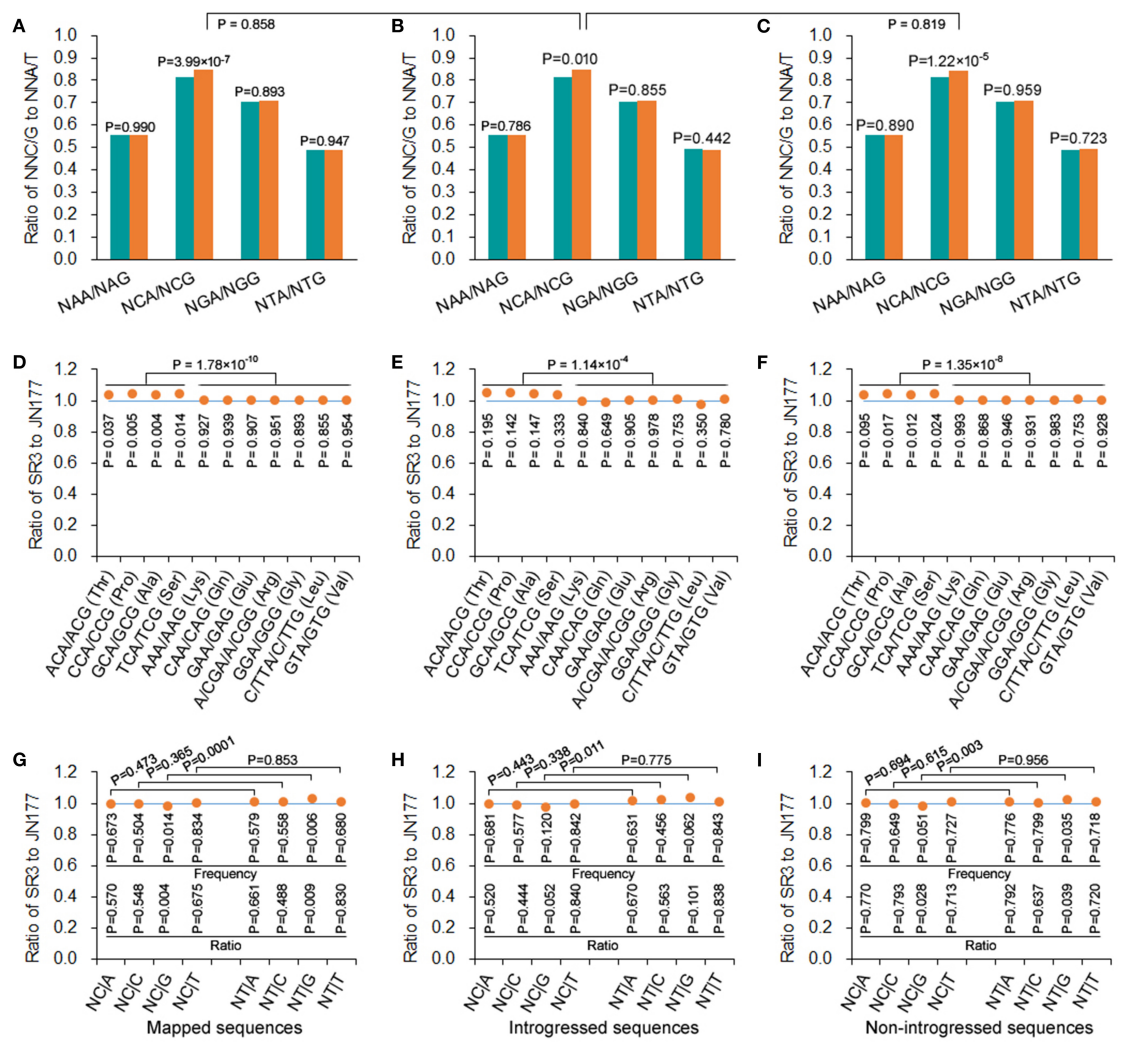
DNA methylation-driven SCUB shift was more pronounced. **(A–C)** The ratio of NNA frequency to NNG frequency. **(D–F)** The ratio of the number of NNA to NNG codons encoding a given amino acid. **(G–I)** The ratio of the frequency of NC|N adjacent codons and NT|N adjacent codons between SR3 and JN177. In **(D)** to **(F)**, the difference in the ratios of Thr, Pro, Ala, and Ser from the ratios of other amino acids was calculated using *t*-test. The other statistical comparison was conducted with chi square (χ^2^) test of fourfold cross-table analysis.

In mapped, introgressed, and non-introgressed sequences, the NCA/NCG ratio (illustrating the conversion of C to T in antisense strand) was higher in SR3 than in JN177 (*P* = 3.99 × 10^−9^, χ^2^ test), but the NGA/NGG, NAA/NAG, and NTA/NTG ratios were comparable between two cultivars (*P* = 0.893–0.990, χ^2^ test) (Figure 4A). On the other hand, we calculated the NT|G and NC|G frequencies to evaluate the effect of C-to-T conversion of sense strand. In mapped sequences, the NC|G frequency of SR3 was significantly lower than JN177's frequency (*P* = 0.014, χ^2^ test), and the ratio of SR3's NC|G frequency to JN177's NC|G frequency was <1 (*P* = 0.004, χ^2^ test); the frequencies of NA|G, NG|G, and NT|G were comparable between SR3 and JN177 (*P* = 0.504–0.834, χ^2^ test), and the ratios of the frequencies were all around 1 (*P* = 0.488–0.830, χ^2^ test) (Figure 4G). Oppositely, the NT|G frequency of SR3 was significantly higher than JN177's frequency (*P* = 0.006, χ^2^ test), and the ratio of SR3's NC|G frequency to JN177's NC|G frequency was more than 1 (*P* = 0.009, χ^2^ test); the frequencies of NA|G, NG|G, and NT|G were similar between SR3 and JN177 (*P* = 0.504–0.834, χ^2^ test), and the ratios of the frequencies were also near to 1 (*P* = 0.488–0.830, χ^2^ test) (Figure 4G). The NT|G/NC|G ratio exhibited a significant difference between JN177 and SR3 (*P* = 0.0001), but NT|A/NC|A, NT|C/NC|C, and NT|T /NC|T ratios were similar (*P* = 0.365–0.853). These results were repeated in introgressed and non-introgressed sequences (Figures 4B,C,H,I). Moreover, the NCA/NCG ratio in introgressed sequences was similar to non-introgressed and mapped sequences (*P* = 0.819 and 0.858, χ^2^ test) (Figures 4A–C); the NT|G/NC|G ratios were also comparable between introgressed sequences and non-introgressed/mapped sequences (Figures 4G–I). These results indicated that DNA methylation affected SCUB in SR3, and the effect appeared to be similar in introgressed and non-introgressed sequences.

To further confirm this effect, A- and G-ending SC pairs encoding a given amino acid sharing the same nucleotides in their first and second positions were analyzed. In mapped sequences, the frequencies of NCA/NCG pairs (encoding alanine, proline, serine, and threonine) of SR3 were higher than those of JN177 (*P* = 0.004–0.037, χ^2^ test), while the frequencies of N(A/G/T)A/N(A/G/T)G pairs (encoding Lys, Gln, Glu, Arg, Gly, Leu, and Val) were similar between SR3 and JN177 (*P* = 0.855–0.954, χ^2^ test) (Figure 4D). The ratios of the frequencies of NCA/NCG of SR3 to JN177 were larger than 1, and significantly larger than those of N(A/G/T)A/N(A/G/T)G pairs (around 1) (*P* = 1.78 × 10^−10^, *t*-test). In introgressed and non-introgressed sequences, SR3 also had higher frequencies of NCA/NCG pairs but comparable frequencies of other pairs in comparison with JN177 (Figures 4E,F).

### SCUB in Allelic Chromosomes and Sub-genomes

**Figure 5:**
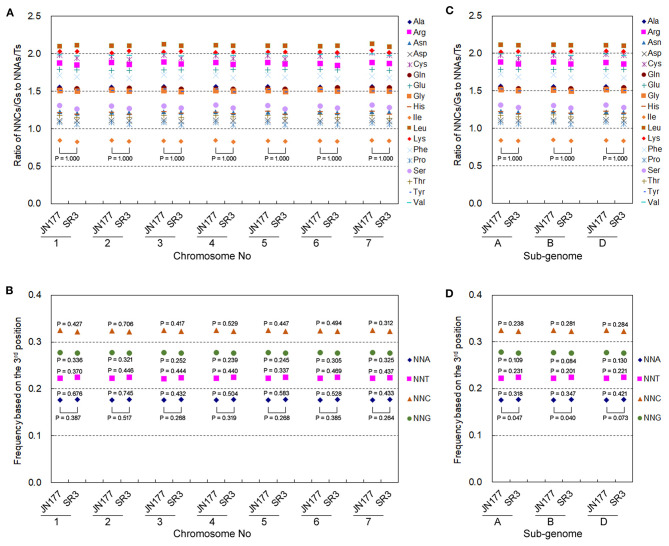
The patterns of SCUB shift based on allelic chromosomes and sub-genomes. **(A,C)** The ratios of the frequencies of NNC/NNG SCs to NNA/NNT SCs in seven groups of allelic chromosomes **(A)** and three sub-genomes **(C)**. NNCs/Gs: SCs with C or G as their final bases; NNAs/Ts: SCs with A or T as their final base. **(B,D)** The total frequency of NNA, NNT, NNC, and NNG codons in seven groups of allelic chromosomes **(B)** and three sub-genomes **(D)**. The total frequency was calculated as the ratio between the amounts number of all SCs ending with A, T, C, or G and the amount of all SCs. The statistical comparison was conducted with chi square (χ^2^) test of fourfold cross-table analysis.

Allohexaploid wheat has seven groups of allelic chromosomes originating from A, B, and D sub-genomes. The genetic variation exhibited different frequencies and patterns among seven allelic chromosomes as well as three sub-genomes in SR3 (Wang et al., 2018), so we further analyzed the heterogeneity of their SCUB alteration. Here, the SC frequencies of 18 amino acids in each of seven allelic chromosomes totally had no statistical difference between JN177 and SR3 (Figure 5A), and the frequencies among seven allelic chromosomes were drastically consistent in both JN177 (CV = 0.001–0.009, Cronbach's alpha = 1.00) and SR3 (CV = 0.001–0.012, Cronbach's alpha = 1.00). The total SCUB frequencies of NNA, NNT, NNC, and NNG in each of seven allelic chromosomes were also similar between JN177, although the difference values were similar to those calculated using all unigenes (Figure 5B). On the other hand, the SC frequencies of 18 amino acids in each of the three sub-genomes were comparable between JN177 and SR3 (Figure 5C), and the consistency of the frequencies among three sub-genomes was also very strong in two cultivars (CV = 0.001–0.007 in JN177 and 0.0003–0.013 in SR3, Cronbach's alpha was 1.00). The total frequencies of NNA, NNT, NNC, and NNG in sub-genomes of SR3 were different from those of JN177 (*P* = 0.040–0.073) (Figure 5D). However, the difference in each of the NNA, NNT, NNC, and NNG frequencies between JN177 and SR3 appeared to be weak. The weaker difference was majorly due to the fewer amounts of SCs for statistical analysis.

## Discussion

Genetic variation serves as a driver of SCUB. Asymmetric somatic hybridization induces high frequency of genome-scale genetic variation such as nucleotide substitutions and indels (Feng et al., 2004; Liu et al., 2007, 2009, 2015; Wang et al., 2015, 2018). Here, we found that the SCUB frequencies exhibited difference between Shanrong 3 (SR3) and Jinan 177 (JN177) (Figure 1), showing that asymmetric somatic hybridization can affect SCUB. Moreover, genetic variation is suffered from selection pressure during plant evolution, and the coding sequence of the gene is under stronger selection pressure than untranslated regions (UTR) (Vinogradov, 2004). We previously found that genetic variation being stably reserved in SR3 is under selection pressure (Wang et al., 2018). SCUB is associated with a balance between mutation, genetic drift, and natural selection (Akashi, 2001; Adams and Wendel, 2005). Thus, the shift of SCUB in the genome of SR3 mirrors the selection pressure of genetic variation induced by asymmetric somatic hybridization.

Genomic shock has proved to induce genetic variation during natural evolution and dipolyploidization of polyploidies (McClintock, 1984; Chen, 2007). Introgression lines are specific polyploidies, but unlike polyploidies, chromosome rearrangement and large fragment deletion that are the force of genomic shock do not usually take place (Xia, 2009). However, our previous studies found that the genomic shock induced by the introgression of exogenous fragments led to the high frequency of genetic variation at the whole-genome scale rather than local chromosomal scale (Wang et al., 2018). Consistently, the shift of SCUB also occurred at the whole-genome scale, and the frequency in chromosome introgressed with and without exogenous fragments was similar (Figure 2), further confirming that the introgression of exogenous fragments predominantly leads to whole genomic shock so that high frequency of genetic variation is induced at the whole-genome scale. The possible causes are as follows: (1) end-joining of fragments as the mechanism of the introgression of donor chromatin segments usually results in point mutations and deletions during repair (Grundy et al., 2014); (2) besides visible fragments by GISH, small invisible exogenous fragments may contribute to the genomic shock.

There has a close association between indel and nucleotide substitution. Nucleotide substitution has higher frequency in flanking sequences of indel than non-flanking sequences (Tian et al., 2008), and substitution frequency appears to increases following the distance decrease to indels (Zhang et al., 2008; Guo et al., 2016). Thus, indel serves as a local “mutator” (Tian et al., 2008; Conrad et al., 2010; De and Babu, 2010; Hollister et al., 2010). In SR3 genome, indel-flanking sequences has higher nucleotide substitution frequency, and the frequency increases close to indels (Wang et al., 2018), showing that indel is also a local “mutator” under the whole genomic shock induced by the introgression of exogenous fragments. Here, SCUB frequency was also altered more strongly in indel-flanking sequences than non-flanking sequences in SR3 genome (Figure 3), consistent with the rule of indel as a local “mutator.” Moreover, the introgression of exogenous fragments performs a local chromosomal shock to promote higher nucleotide substitution frequency at 5′-side flanking sequences of indels in chromosomes with exogenous fragments than that in chromosomes without exogenous fragments (Wang et al., 2018). However, the SCUB frequency of two-side flanking sequences of indels was similar between chromosomes with and without exogenous fragments (Figure 3), indicating the difference in the patterns of nucleotide substitution and SCUB.

The alteration of cytosine methylation, a kind of variation induced by genomic shock, often occurs in the genomes of allopolyploidies (Comai, 2000; Shaked et al., 2001; Kashkush et al., 2002, 2003) and newly synthesized allohexaploid wheat (Shaked et al., 2001). Similarly, asymmetric somatic hybridization also alters cytosine methylation patterns in wheat (Wang M. et al., 2014; Liu et al., 2015). Methylated cytosines can be converted to thymine (Ossowski et al., 2010), and therefore is a major source of SNP formation (C → T, and G → A in complementary strand) (Laird, 2010). We previously proved that epigenetic modification-mediated nucleotide substitution is one of the major forces of genetic variation induced by asymmetric somatic hybridization in wheat (Wang et al., 2018). Here, DNA methylation was also closely associated with SCUB shift in SR3 genome (Figure 4). In line with the association of DNA methylation and SCUB differentiation during plant evolution (Qin et al., 2013; Qi et al., 2015; Xu et al., 2015), it could be concluded that epigenetic variation may play crucial roles in genetic variation induced by genomic shock, and there have been a close association between genetic and epigenetic variation.

As two major types of genetic variation, both nucleotide substitution and small indels suffer from selection pressure (McNally et al., 2006). In wheat, asymmetric somatic hybridization induces similar genetic variation among non-allelic chromosomes that suffer from similar selection pressure, but different genetic variation among three sub-genomes that suffer from different selection pressure, showing that the induction of genetic variation is a non-random but regulatory process (Wang et al., 2018). However, the SCUB frequencies in seven non-allelic as well as three sub-genomes chromosomes exhibited similar difference values of SCUB frequencies between SR3 and JN177, but the difference values were similar to those calculated using all unigenes (Figure 5), indicating the consistency and specificity between nucleotide substitution and SCUB induced by asymmetric somatic hybridization.

SR3 is a salt-tolerant cultivar with stronger salt tolerance capacity and higher yield than JN177 (Xia et al., 2003). Our previous work found that there was a remarkable difference in transcriptomic and proteomic profiles between these two cultivars under normal and saline conditions (Wang et al., 2008; Peng et al., 2009; Liu et al., 2012), which is partially attributed to the whole-genome scale genetic and epigenetic variation in SR3, including transposon activation, DNA methylation, as well as indels and nucleotide substitution in the promoters. For instance, the variation in promoter of a salt-tolerant associated gene *TaCHP* offers its transcription increase in SR3. Besides, large-scale EST comparison showed that a set of genes possessing genetic variation in CDS exhibits differential transcriptional profile between SR3 and Jn177. More importantly, the variation in CDS of some salt-tolerant associated genes alters the function of encoding products in salt response, of which the amino acid substitutions in TaSRO1, the putative salt-tolerant major QTL candidate gene, and TaSOD2 enhances their PARP and SOD activities, respectively. On the other hand, given the role of SCs in transcription efficiency, mRNA stability, translational efficiency, and accuracy and other aspects (Marais et al., 2001; Warnecke and Hurst, 2007; Zhang et al., 2009; Tuller et al., 2010; Presnyak et al., 2015), the SCUB shift may also account for the superior salt tolerance of SR3. Although there are no good or established tests for functional SCs so far, it is worthy of studying the biological performance in asymmetric somatic hybridization, natural evolution, and other events.

## Conclusions

This work firstly addressed the patterns of asymmetric somatic hybridization-induced SCUB shift. Asymmetric somatic hybridization induced a whole-genome scale shift of SCUB, and introgressed exogenous fragments did not induce a stronger shift of SCUB in introgressed chromosomes, showing that SCUB was shifted *via* whole genomic shock rather than local chromosomal shock. Asymmetric somatic hybridization induced indels that promoted SCUB shift in flanking sequences. DNA methylation was a driver of SCUB shift, indicating the complicated association between genetic and epigenetic variation induced by asymmetric somatic hybridization.

## Data Availability Statement

Publicly available datasets were analyzed in this study. This data can be found here: Sequences used for analysis in this work were submitted to Genbank (Accession number: JZ881292–JZ892704).

## Author Contributions

MW and GX designed the research. MW, WX, and CL performed the experiments. MW, YiL, YaL, and YW analyzed the data. MW and GX wrote the paper. All authors read and approved the final manuscript.

## Conflict of Interest

The authors declare that the research was conducted in the absence of any commercial or financial relationships that could be construed as a potential conflict of interest.

## Supplementary Material

Supplementary Figure 1 The statistical of codons of unigenes. **(A)** The length of total unigenes as well as their CDS and UTR. **(B)** The codon amounts in CDS.

Supplementary Figure 2 The frequencies of amino acid encoding codons.

Supplementary Figure 3 SCUB frequencies in chromosomes introgressed with and without exogenous fragments using aligned sequences between JN177 and SR3. **(A)** The ratios of the frequencies of NNC/NNG SCs to NNA/NNT SCs. NNCs/Gs: SCs with C or G as their final bases; NNAs/Ts: SCs with A or T as their final base. **(B)** The total frequency of NNA, NNT, NNC, and NNG codons. The total frequency was calculated as the ratio between the amounts number of all SCs ending with A, T, C, or G and the amount of all SCs. Total: all unigenes; Mapped: unigenes mapped to chromosomes; Introgressed: unigenes from chromosomes introgressed with exogenous fragments; Non-introgresed: unigenes from chromosomes without exogenous fragments. The statistical comparison was conducted with chi square *(*χ2) test of fourfold cross-table analysis.

Supplementary Table 1 The statistical analysis of SCUB frequencies of 18 amino acids.

Supplementary Table 2 The statistical analysis of SCUB frequencies of 18 amino acids in chromosomes with and without exogenous fragments.

Supplementary Table 3 The consistency of SCUB frequencies of 18 amino acids.

Supplementary Table 4 The statistical analysis of SCUB frequencies of 18 amino acids in chromosomes with and without exogenous fragments based on aligned sequences.

Supplementary Table 5 The statistical analysis of total SCUB frequencies between JN177 and SR3.
