# Supplementary material for: Asymmetric Somatic Hybridization Affects Synonymous Codon Usage Bias in Wheat
Source: Front Genet. 2021 Jun 11;12:682324. doi: 10.3389/fgene.2021.682324 (PMC8226224; doi:10.3389/fgene.2021.682324)
Supplement: Supplementary Figure 1 — The statistical of codons of unigenes. (A) The length of total unigenes as well as their CDS and UTR. (B) The codon amounts in CDS. [file Data_Sheet_1.PDF]

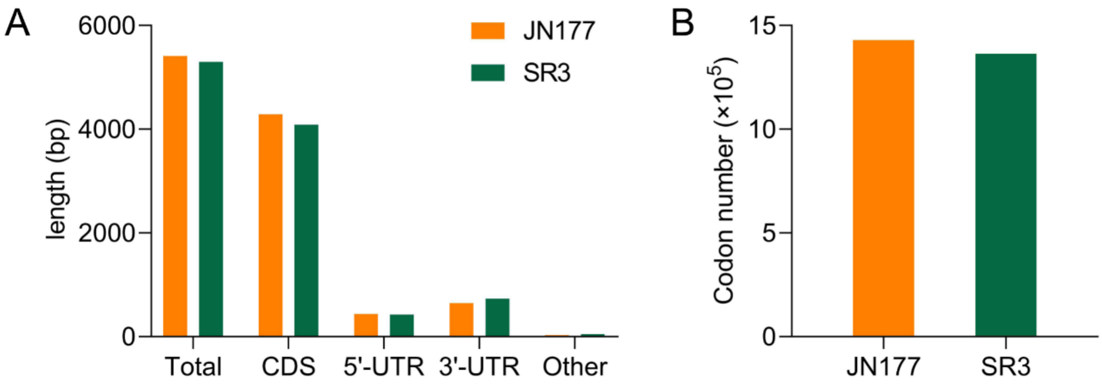


Supplemental Figure S1

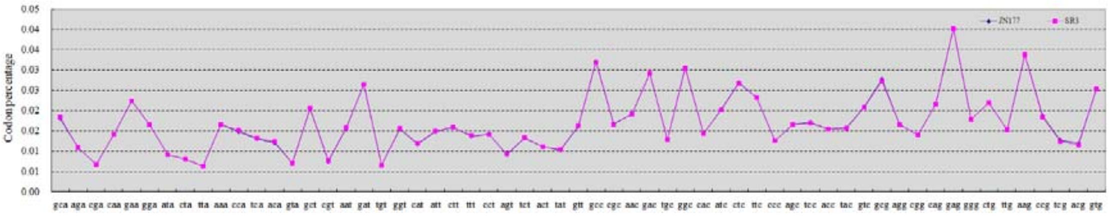


Supplemental Figure S2

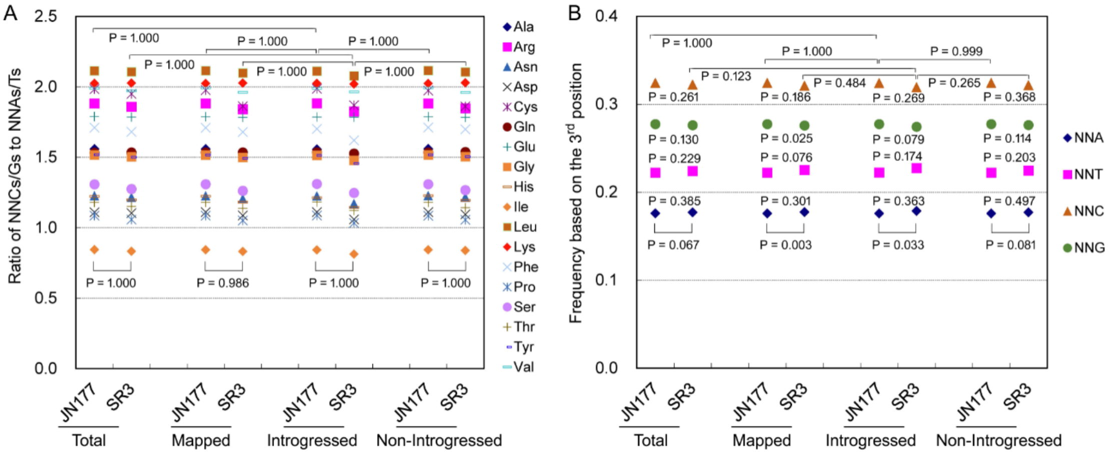


Supplemental Figure S3

Supplemental Table S1. The statistical analysis of SCUB frequencies of 18 amino acids.

| | χ2 test of SC | CV of SCs | | χ2 test of NNC/G vs NNA/T | CV of NNC/G vs NNA/T |
|---|---|---|---|---|---|
| | | JN177 | SR3 | | |
| Ala | 0.009 | 0.265 | 0.254 | 0.003 | 0.017 |
| Arg | 0.566 | 0.365 | 0.359 | 0.257 | 0.008 |
| Asn | 0.463 | 0.144 | 0.137 | 0.463 | 0.007 |
| Asp | 0.592 | 0.073 | 0.069 | 0.592 | 0.004 |
| Cys | 0.419 | 0.466 | 0.456 | 0.419 | 0.011 |
| Gln | 0.988 | 0.299 | 0.298 | 0.988 | 1.45E-04 |
| Glu | 0.998 | 0.400 | 0.400 | 0.998 | 2.11E-05 |
| Gly | 0.672 | 0.353 | 0.347 | 0.363 | 0.006 |
| His | 0.122 | 0.142 | 0.126 | 0.122 | 0.017 |
| Ile | 0.404 | 0.375 | 0.370 | 0.233 | 0.010 |
| Leu | 0.995 | 0.501 | 0.499 | 0.658 | 0.003 |
| Lys | 0.984 | 0.480 | 0.480 | 0.984 | 1.63E-04 |
| Phe | 0.179 | 0.371 | 0.360 | 0.179 | 0.013 |
| Pro | 0.028 | 0.174 | 0.165 | 0.007 | 0.019 |
| Ser | 0.012 | 0.212 | 0.206 | 0.002 | 0.019 |
| Thr | 0.086 | 0.156 | 0.153 | 0.024 | 0.017 |
| Try | 0.437 | 0.291 | 0.283 | 0.437 | 0.008 |
| Val | 0.633 | 0.450 | 0.448 | 0.316 | 0.007 |

Supplemental Table S2. The statistical analysis of SCUB frequencies of 18 amino acids in chromosomes with and without exogenous fragments.

| Amino acid | JN177 *vs* SR3 | | | | Introgressed *vs* Non-introgressed | | Introgressed *vs* Mapped | | Introgressed *vs* Total | |
|---|---|---|---|---|---|---|---|---|---|---|
| | Total | Mapped | Introgressed | Non-introgressed | JN177 | SR3 | JN177 | SR3 | JN177 | SR3 |
| Ala | 0.009 | 0.023 | 0.527 | 0.060 | 0.992 | 0.997 | 0.996 | 0.998 | 0.996 | 0.998 |
| Arg | 0.566 | 0.522 | 0.934 | 0.696 | 0.990 | 0.963 | 0.997 | 0.988 | 0.996 | 0.997 |
| Asn | 0.463 | 0.573 | 0.713 | 0.660 | 0.925 | 0.809 | 0.941 | 0.850 | 0.943 | 0.903 |
| Asp | 0.592 | 0.570 | 0.972 | 0.528 | 0.870 | 0.820 | 0.898 | 0.858 | 0.936 | 0.856 |
| Cys | 0.419 | 0.407 | 0.780 | 0.427 | 0.996 | 0.835 | 0.997 | 0.870 | 0.999 | 0.916 |
| Gln | 0.988 | 0.939 | 0.649 | 0.868 | 0.781 | 0.691 | 0.828 | 0.755 | 0.786 | 0.746 |
| Glu | 0.998 | 0.907 | 0.905 | 0.946 | 0.930 | 0.851 | 0.945 | 0.883 | 0.935 | 0.813 |
| Gly | 0.672 | 0.770 | 0.867 | 0.897 | 0.982 | 0.985 | 0.991 | 0.993 | 0.988 | 0.994 |
| His | 0.122 | 0.193 | 0.482 | 0.272 | 0.923 | 0.989 | 0.940 | 0.992 | 0.997 | 0.988 |
| Ile | 0.404 | 0.473 | 0.446 | 0.734 | 0.841 | 0.821 | 0.899 | 0.886 | 0.896 | 0.881 |
| Leu | 0.995 | 0.992 | 0.964 | 0.987 | 0.953 | 0.996 | 0.984 | 0.999 | 0.956 | 0.984 |
| Lys | 0.984 | 0.927 | 0.840 | 0.993 | 0.975 | 0.823 | 0.981 | 0.861 | 0.950 | 0.852 |
| Phe | 0.179 | 0.224 | 0.609 | 0.269 | 0.793 | 0.898 | 0.837 | 0.920 | 0.776 | 0.865 |
| Pro | 0.028 | 0.043 | 0.491 | 0.119 | 0.992 | 0.980 | 0.996 | 0.990 | 0.998 | 0.987 |
| Ser | 0.012 | 0.053 | 0.773 | 0.130 | 0.999 | 0.997 | 1.000 | 0.999 | 0.999 | 1.000 |
| Thr | 0.086 | 0.158 | 0.524 | 0.316 | 0.916 | 0.980 | 0.957 | 0.990 | 0.963 | 0.995 |
| Tyr | 0.437 | 0.486 | 0.842 | 0.363 | 0.692 | 0.633 | 0.756 | 0.708 | 0.737 | 0.713 |
| Val | 0.633 | 0.722 | 0.983 | 0.717 | 0.978 | 0.988 | 0.989 | 0.994 | 0.990 | 0.981 |

Supplemental Table S3. The consistency of SCUB frequencies of 18 amino acids.

| | Mean of SCUB frequency | | | SD of SCUB frequency | | | CV of SCUB frequency | | |
|---|---|---|---|---|---|---|---|---|---|
| | JN177 | SR3 | Total | JN177 | SR3 | Total | JN177 | SR3 | Total |
| Ala | 1.558 | 1.521 | 1.540 | 0.003 | 0.001 | 0.002 | 0.002 | 4.99E-04 | 0.002 |
| Arg | 1.880 | 1.857 | 1.869 | 0.007 | 0.008 | 0.002 | 0.004 | 0.004 | 0.001 |
| Asn | 1.226 | 1.215 | 1.221 | 0.001 | 0.003 | 0.001 | 0.001 | 0.002 | 0.001 |
| Asp | 1.110 | 1.104 | 1.107 | 0.001 | 0.002 | 4.42E-04 | 0.001 | 0.002 | 3.99E-04 |
| Cys | 1.982 | 1.951 | 1.967 | 1.37E-04 | 0.006 | 0.002 | 6.94E-05 | 0.003 | 0.001 |
| Gln | 1.532 | 1.537 | 1.535 | 0.005 | 0.006 | 0.001 | 0.003 | 0.004 | 4.68E-04 |
| Glu | 1.787 | 1.785 | 1.786 | 0.001 | 0.003 | 3.24E-04 | 0.001 | 0.002 | 1.81E-04 |
| Gly | 1.517 | 1.502 | 1.509 | 0.004 | 0.002 | 0.001 | 0.003 | 0.002 | 0.001 |
| His | 1.223 | 1.196 | 1.209 | 0.002 | 2.14E-04 | 0.002 | 0.001 | 1.79E-04 | 0.002 |
| Ile | 0.844 | 0.832 | 0.838 | 0.002 | 0.002 | 0.001 | 0.002 | 0.003 | 0.001 |
| Leu | 2.111 | 2.108 | 2.109 | 0.014 | 0.003 | 0.001 | 0.006 | 0.001 | 0.001 |
| Lys | 2.025 | 2.028 | 2.027 | 0.001 | 0.004 | 4.20E-04 | 0.001 | 0.002 | 2.07E-04 |
| Phe | 1.709 | 1.680 | 1.694 | 0.005 | 0.003 | 0.002 | 0.003 | 0.002 | 0.001 |
| Pro | 1.087 | 1.057 | 1.072 | 0.002 | 0.001 | 0.002 | 0.001 | 0.001 | 0.002 |
| Ser | 1.309 | 1.275 | 1.292 | 0.003 | 0.002 | 0.002 | 0.002 | 0.002 | 0.002 |
| Thr | 1.181 | 1.151 | 1.166 | 0.003 | 0.002 | 0.002 | 0.003 | 0.002 | 0.002 |
| Tyr | 1.515 | 1.502 | 1.509 | 0.007 | 0.009 | 0.001 | 0.005 | 0.006 | 0.001 |
| Val | 1.989 | 1.970 | 1.980 | 0.004 | 0.002 | 0.001 | 0.002 | 0.001 | 0.001 |

Supplemental Table S4. The statistical analysis of SCUB frequencies of 18 amino acids in chromosomes with and without exogenous fragments based on aligned sequences.

| Amino acid | JN177 *vs* SR3 | | | | Introgressed *vs* Non-introgressed | | Introgressed *vs* Mapped | | Introgressed *vs* Total | |
|---|---|---|---|---|---|---|---|---|---|---|
| | Aligned | Mapped | Introgressed | Non-introgressed | JN177 | SR3 | JN177 | SR3 | JN177 | SR3 |
| Ala | 0.120 | 0.097 | 0.237 | 0.214 | 0.938 | 0.490 | 0.951 | 0.587 | 0.952 | 0.474 |
| Arg | 0.487 | 0.288 | 0.465 | 0.417 | 1.000 | 0.729 | 1.000 | 0.785 | 0.997 | 0.582 |
| Asn | 0.737 | 0.541 | 0.436 | 0.790 | 0.975 | 0.418 | 0.981 | 0.524 | 0.987 | 0.404 |
| Asp | 0.783 | 0.327 | 0.308 | 0.579 | 1.000 | 0.378 | 1.000 | 0.488 | 0.979 | 0.254 |
| Cys | 0.635 | 0.137 | 0.458 | 0.198 | 0.922 | 0.948 | 0.939 | 0.959 | 0.979 | 0.505 |
| Gln | 0.959 | 0.996 | 0.920 | 0.951 | 0.984 | 0.884 | 0.987 | 0.909 | 0.967 | 0.906 |
| Glu | 0.923 | 0.934 | 0.991 | 0.929 | 0.979 | 0.984 | 0.983 | 0.988 | 0.965 | 0.996 |
| Gly | 0.636 | 0.545 | 0.548 | 0.719 | 0.949 | 0.576 | 0.960 | 0.660 | 0.950 | 0.566 |
| His | 0.479 | 0.294 | 0.388 | 0.471 | 0.830 | 0.431 | 0.866 | 0.535 | 0.878 | 0.383 |
| Ile | 0.545 | 0.587 | 0.479 | 0.819 | 0.982 | 0.458 | 0.986 | 0.560 | 0.972 | 0.541 |
| Leu | 0.843 | 0.674 | 0.667 | 0.808 | 0.946 | 0.665 | 0.958 | 0.733 | 0.976 | 0.634 |
| Lys | 0.961 | 0.953 | 0.966 | 0.928 | 0.989 | 0.919 | 0.992 | 0.936 | 0.998 | 0.932 |
| Phe | 0.491 | 0.537 | 0.401 | 0.810 | 0.904 | 0.320 | 0.924 | 0.434 | 0.916 | 0.421 |
| Pro | 0.175 | 0.134 | 0.260 | 0.276 | 0.966 | 0.546 | 0.974 | 0.634 | 0.988 | 0.498 |
| Ser | 0.131 | 0.050 | 0.200 | 0.125 | 0.965 | 0.624 | 0.972 | 0.700 | 0.950 | 0.456 |
| Thr | 0.240 | 0.120 | 0.290 | 0.234 | 0.957 | 0.662 | 0.967 | 0.731 | 0.960 | 0.513 |
| Tyr | 0.685 | 0.637 | 0.572 | 0.846 | 0.962 | 0.555 | 0.970 | 0.642 | 0.948 | 0.571 |
| Val | 0.646 | 0.461 | 0.770 | 0.496 | 0.963 | 0.953 | 0.971 | 0.963 | 0.991 | 0.896 |

Supplemental Table S5. The statistical analysis of total SCUB frequencies between JN177 and SR3.

| | | Whole | | 5'-side | | 3'-side | | Two-sides | | Remote | |
|---|---|---|---|---|---|---|---|---|---|---|---|
| | | Ratio | P | Ratio | P | Ratio | P | Ratio | P | Ratio | P |
| Mapped | NNA | 1.009 | 0.186 | 1.024 | 0.413 | 1.022 | 0.457 | 1.023 | 0.269 | 1.007 | 0.310 |
| | NNT | 1.014 | 0.025 | 1.039 | 0.152 | 1.029 | 0.285 | 1.034 | 0.077 | 1.012 | 0.081 |
| | NNC | 0.991 | 0.076 | 0.973 | 0.241 | 0.980 | 0.382 | 0.977 | 0.148 | 0.992 | 0.169 |
| | NNG | 0.994 | 0.301 | 0.985 | 0.533 | 0.986 | 0.571 | 0.986 | 0.400 | 0.995 | 0.422 |
| Introgressed | NNA | 1.016 | 0.269 | 1.024 | 0.698 | 1.022 | 0.718 | 1.023 | 0.596 | 0.583 | 0.324 |
| | NNT | 1.023 | 0.079 | 1.038 | 0.501 | 1.030 | 0.590 | 1.034 | 0.391 | 0.378 | 0.118 |
| | NNC | 0.985 | 0.174 | 0.974 | 0.585 | 0.980 | 0.676 | 0.977 | 0.495 | 0.507 | 0.229 |
| | NNG | 0.989 | 0.363 | 0.985 | 0.765 | 0.985 | 0.764 | 0.985 | 0.672 | 0.682 | 0.414 |
| Non-Introgressed | NNA | 1.007 | 0.368 | 1.024 | 0.471 | 1.022 | 0.515 | 1.023 | 0.332 | 1.005 | 0.540 |
| | NNT | 1.011 | 0.114 | 1.039 | 0.205 | 1.028 | 0.356 | 1.034 | 0.122 | 1.009 | 0.259 |
| | NNC | 0.992 | 0.203 | 0.973 | 0.300 | 0.980 | 0.443 | 0.977 | 0.202 | 0.994 | 0.366 |
| | NNG | 0.996 | 0.203 | 0.985 | 0.585 | 0.987 | 0.630 | 0.986 | 0.467 | 0.997 | 0.366 |
